# Atypical Giant Hydatid Cyst at the Thoracic Wall Causing Bone and Soft Tissue Destruction: Report of a Case

**DOI:** 10.5812/ircmj.10584

**Published:** 2013-06-05

**Authors:** Arife Zeybek, Abdullah Erdoğan, Şirin Akdeniz, Gökçen Kenar, Levent Dertsiz, Abid Demircan

**Affiliations:** 1 School of Medicine, Akdeniz University, Thoracic Surgery Clinics, Antalya, Turkey; 2 School of Medicine, Mugla Sıtkı Koçman University, Thoracic Surgery Clinics, Muğlam, Turkey

**Keywords:** Echinococcosis, Atypical, Thoracic Wall

## Abstract

Hydatid cyst is a zoonotic disease that is common in the Mediterranean region. Thoracic wall, rib or extrapulmonary intrathoracic localization of the cysts is very rare. Giant extrapulmonary intrathoracic hydatid cysts can lead to both diagnostic and treatment difficulties and can be confused with tumor. We present a case of a hydatid cyst with thoracic wall involvement mimicking tumor. We confirmed diagnosis only by surgical exploration and histopathological examination because radiology is not conclusive .Surgical treatment involved the total extirpation of cyst together wide debridement and resection of affected tissue. Primary thoracic wall closures were performed. In thoracic wall localization of cyst, post-operative course of albendazole for 6 weeks associated with surgery can help in sterilizing the cyst and reduce the recurrence rate. In this article, we presented a thoracic wall hydatidosis which is very uncommon asymptomatic presentation of hydatid cyst disease with its surgical management.

## 1. Background

Hydatid cyst is a parasitic infection that is more common in countries along the Mediterranean Sea coast than in other European countries (1). Humans are not involved in the natural cycle of the cyst; they become accidentally involved in the cycle via oral ingestion of the cystic form of the parasite (2). The orally ingested cyst unfolds in the duodenum and initially spreads to the liver via the vena porta via a hematogenous or sometimes lymphogenous route, and then to the lungs via the venous system; from the lungs it can potentially spread to any organ via the arterial system (3). The parasite can localize in any organ in the body with an arterial blood supply (rarely the brain, bones, teeth, nails, endothelium), but the most common localization is the liver (55-60% of cases), followed by the lungs (25-30% of cases); localization to other organs occurs in approximately 5% of cases (4-6).

A giant hydatid cyst with primary chest wall localization and intrathoracic cavity extension that causes atelectasis in the lungs, and widespread necrosis and destruction in the lateral regions of the ribs and soft tissue of the chest wall (muscle, fascia, and fat tissue) is an extremely rare phenomenon. Herein we report such a case, in which the cyst exhibited the same density as that of the chest wall soft tissue in radiological images and could not be definitely identified as a cystic mass via thoracic computerized tomography (CT) or thoracic ultrasonography (US), as it was observed as a soft tissue density.

## 2. Case Presentation

The patient is living in the rural part of Antalya province in Turkey. She is a housewife. The patient was admitted to our clinic in 2010. A 65-year-old female presented due to a 5-6 month history of thoracic and neck pain. The patient felt the pain in the right anterior region of the chest and shoulder, and the neck, reporting that the pain was more severe with deep inhalation. She also described lumps in her neck and anterior chest wall that had become apparent during the previous week. Anamnesis did not include any relevant signs or symptoms that could account for her present condition. Physical examination showed that her right hemithorax was contributing less to inspiration and that there were stiff masses with irregular borders and the consistency of soft tissue in the anterior chest wall. There was minimal venous distension in the right side of the neck.

**Figure 1.**
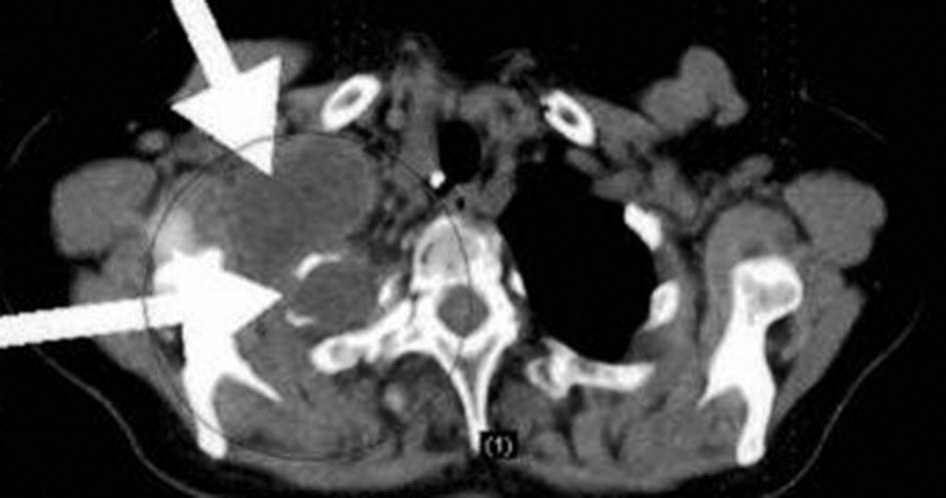
Preoperative PET/CT Image

**Figure 2.**
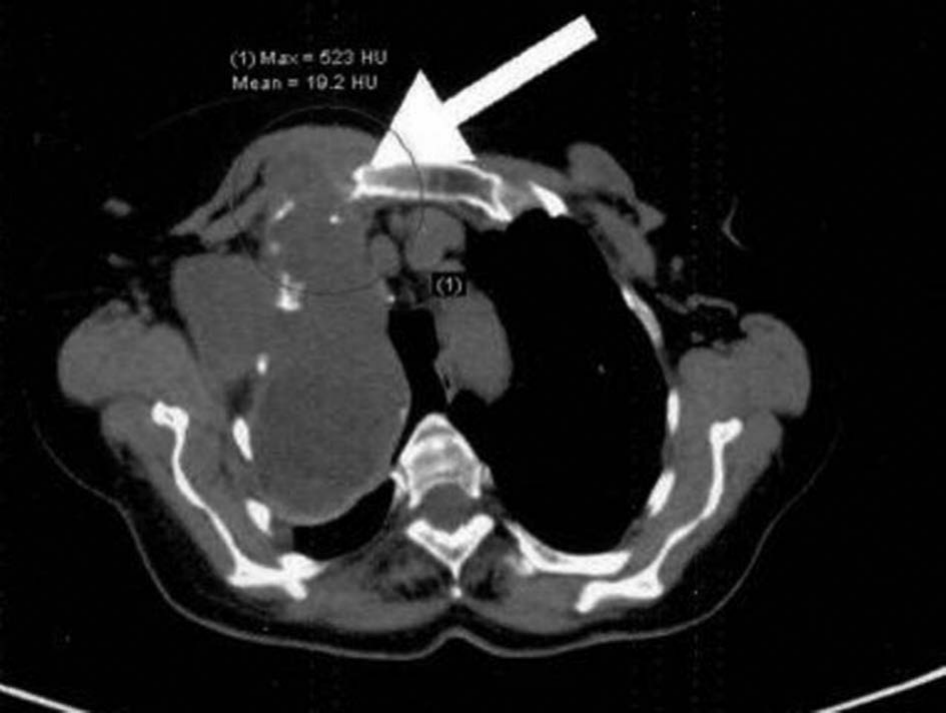
Preoperative Thoracic CT Image

The patient’s laboratory findings were within normal limits. There were some important radiological findings. Thoracic CT showed tumoral masses with giant soft tissue consistency measuring 10 × 15 × 10 cm in total. These masses were in the chest wall and extended to the skin surface. They had nearly filled up the intrathoracic superior chest cavity and caused atelectasis of the lungs due to obstruction, and the density of the masses was similar to that of the chest wall soft tissue. They consisted of multiple masses that conjugated in some parts of the thoracic wall and formed conglomerate. There was also lymphadenopathies 1-1.5 cm in diameter in the mediastinum (Figures 1 and 2). Whole-body positron emission tomography (PET)/CT showed that the lesions noted via thoracic CT were not overly active, and that their max SUV levels were between 3 and 5, and that similarly the mediastinal lymphadenopathies did not exhibit high max SUV levels.

**Figure 3.**
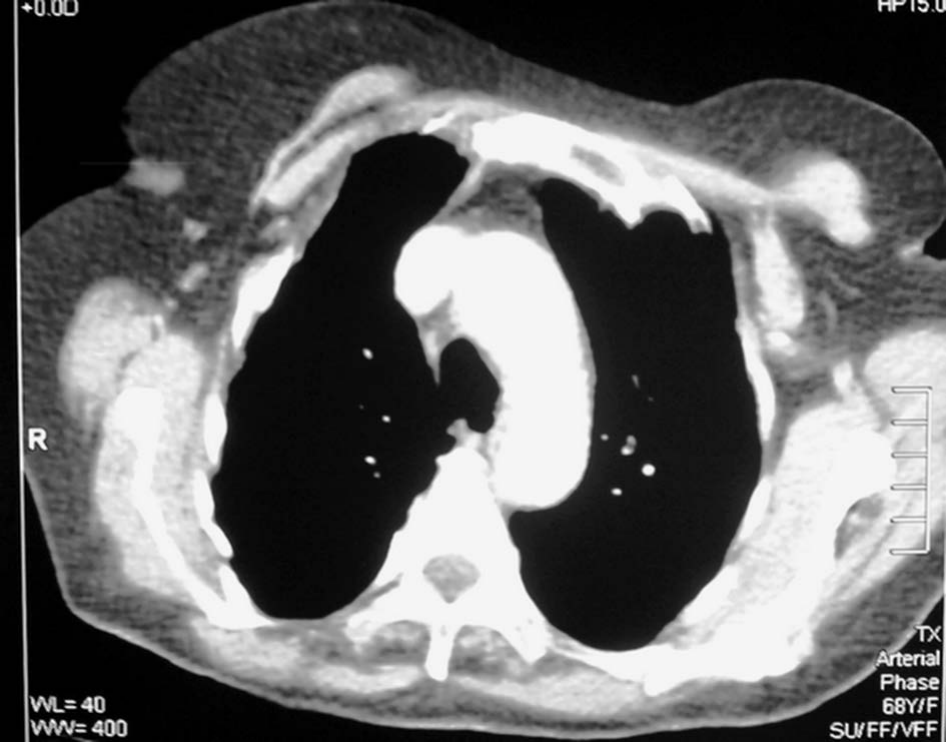
Postoperative Thoracic CT Image in Third Year

The fact that the lesions did not fluctuate on palpation during physical examination and that there was soft tissue density readings on CT, as well as the clinical presentation raised the suspicion of a possible chest wall tumor. As a result, transthoracic fine needle aspiration biopsy was performed; however, cytological examination of the specimen showed benign cytological findings rather than malign. The patient underwent an open biopsy for diagnostic purposes. During the thoracic exploration performed under general anesthesia it was seen that the lesions were cystic structures of high density. The exploration was widened and all cystic structures of the thoracic wall and intrathoracic region were drained. When the thoracic wall was opened during the procedure muscle and soft tissue of the thoracic wall, and anterolateral regions of the 2nd, 3rd and 4th ribs were observed to be entirely necrotic and destroyed, and as such it was easy to enter the thoracic cage. After all the cystic lesions were removed, it was noted that the atelectatic lung in the intrathoracic region easily expanded and filled the chest cavity. After the chest wall regions that were necrotic and destroyed were cleared away, the thoracic cage was closed primarily. 2gr/day of first generation cephalosporins for 10 day and 15-20 mg/kg/day of albendazole for 4 weeks was administered. There weren’t any complications during the postoperative period and the patient was discharged at postoperative 10th day in a state of complete recovery. The postoperative histopathological examination report was hydatid cyst. During routine postoperative follow-up there weren’t any complications or signs of recurrence. At the time this report was prepared the patient was in her third postoperative year and was symptom free (Figure 3).

## 3. Discussion

Echinococcosis/hydatidosis is a zoonotic disease in which humans and herbivorous animals are infected as intermediate hosts. In the Mediterranean region the cause of hydatidosis is usually Echinococcus granulosus (2). It is common in Pakistan, India, Chile, Brazil, North Africa, Bulgaria, Yugoslavia, and the Mediterranean region. The prevalence in Turkey is 3.4 in 100.000 (3). It is more common in people that work with livestock, and is more common in women than men. It is predominantly observed in the liver and lungs, but can localize to any tissue in the body.

Hydatid cysts can be encountered in all tissues and organs, but occur in the chest wall, ribs, and sternum in only 0.9-2% of cases (4); intrathoracic extrapulmonary hydatid cyst occurs in 7.4% of cases (5, 6). The most common symptom of pulmonary parenchyma hydatid cyst is chest pain; however, hydatid cysts in the diaphragm, pleura, mediastinum, pericardium, myocardium, and fissure areas, and intrathoracic extrapulmonary localization can manifest with multiple symptoms due to the pressure they exert on vital organs (7, 8). The hydatid cyst in the presented patient was localized primarily to the chest wall and extended to the intrathoracic extrapulmonary region. During preoperative evaluation it exhibited the signs and radiological characteristics of a chest wall tumor with chest wall invasion. Definitive diagnosis in the presented patient was not possible based on preoperative biochemical, radiological, and interventional investigations. Diagnosis is more difficult in cases of complicated extrapulmonary intrathoracic hydatid cysts due to their density. As in the presented patient, in cases of complicated hydatid cysts with primary chest wall localization and intrathoracic dissemination, radiological techniques are insufficient for diagnosis, and surgical intervention increases the risk of perforation and dissemination to surrounding tissues if the cyst is alive during the surgery. In such cases of hydatidosis, clinical experience is crucial for diagnosis and treatment. In symptomatic and tolerable patients, thoracotomy is the most reliable diagnostic and treatment approach when preoperative biochemical, radiological, and clinical examinations have not yielded a diagnosis (9, 10).

In contrast to pulmonary hydatid cysts, hydatid cysts that cause intrathoracic extrapulmonary rib destruction require complete surgical treatment consisting of cystectomy, wide resection, and reconstruction of pericystic and neighboring tissues, rather than the cystotomy capitonnage technique. Mortality and morbidity both increase in cases of giant complicated hydatid cysts with intrathoracic extrapulmonary localization. Cystectomy was performed in the presented patient and pericystic tissues were resected and debrided. During routine postoperative follow-up there weren’t any complications or signs of recurrence.

## 4. Conclusions

Hydatid cysts can involve all the thoracic structures, primarly in the thoracic wall localization is rare. For patients in whom a thoracic wall mass is detected, especially those living areas where hydatid cyst is endemic, it should be considered in the initial diagnosis. In these cases, surgical treatment combined with medical treatment given during the post-operative course produces succesfull early and late period result.
